# A Swelling in the Mouth in a Chronic Hemodialysis Patient

**DOI:** 10.1155/2016/4970702

**Published:** 2016-10-09

**Authors:** Arnaud Devresse, Alexandros Raptis, Anne-Sophie Claes, Laura Labriola

**Affiliations:** ^1^Department of Nephrology, Cliniques Universitaires Saint-Luc, Université Catholique de Louvain, Av. Hippocrate 10, 1200 Brussels, Belgium; ^2^Department of Pathology, Cliniques Universitaires Saint-Luc, Université Catholique de Louvain, Av. Hippocrate 10, 1200 Brussels, Belgium; ^3^Department of Medical Imaging, Cliniques Universitaires Saint-Luc, Université Catholique de Louvain, Av. Hippocrate 10, 1200 Brussels, Belgium

## Abstract

Oral manifestations of severe secondary hyperparathyroidism include maxillary and mandibular deformities, brown tumors, dental abnormalities, and metastatic calcification of soft tissues. We report on a chronic hemodialysis (HD) woman with severe, uncontrolled secondary hyperparathyroidism and a painful, nontender mass in the floor of her mouth. The most likely clinical diagnosis was a bone tumoral lesion of the oral cavity, secondary to renal osteodystrophy. Unexpectedly, pathological examination showed characteristic features of ossifying fibroma (OF) of the jaw, a rare, benign fibroosseous lesion characterized by the replacement of normal bone by collagen and fibroblasts containing varying amounts of mineralized substance. The occurrence of an OF in chronic HD patients is exceptional. Differential diagnosis must be made with bone tumoral lesions secondary to renal osteodystrophy. Surgical removal is the treatment of choice. The pathogenesis of OF in the setting of secondary hyperparathyroidism remains unknown. Parathyroidectomy may not be necessary to avoid OF recurrence after surgical removal.

## 1. Background

The impact of secondary hyperparathyroidism on the oral cavity includes maxillary and mandibular deformities, dental abnormalities, and metastatic calcification of soft tissues. Rarely, renal osteodystrophy may present as a solitary tumor in the mouth. We report on a chronic hemodialysis (HD) patient presenting a swelling in the floor of her mouth. After ruling out bone metastatic lesions, renal osteodystrophy in the context of severe secondary hyperparathyroidism was the most likely diagnosis. However, pathological examination showed characteristic features of ossifying fibroma (OF) of the jaw, a benign neoplasm composed of fibrocellular tissue and mineralized material. OF has been exceptionally documented in HD patients and may be clinically similar to bone lesions secondary to severe uncontrolled hyperparathyroidism.

## 2. Case Report

**Figure 1:**
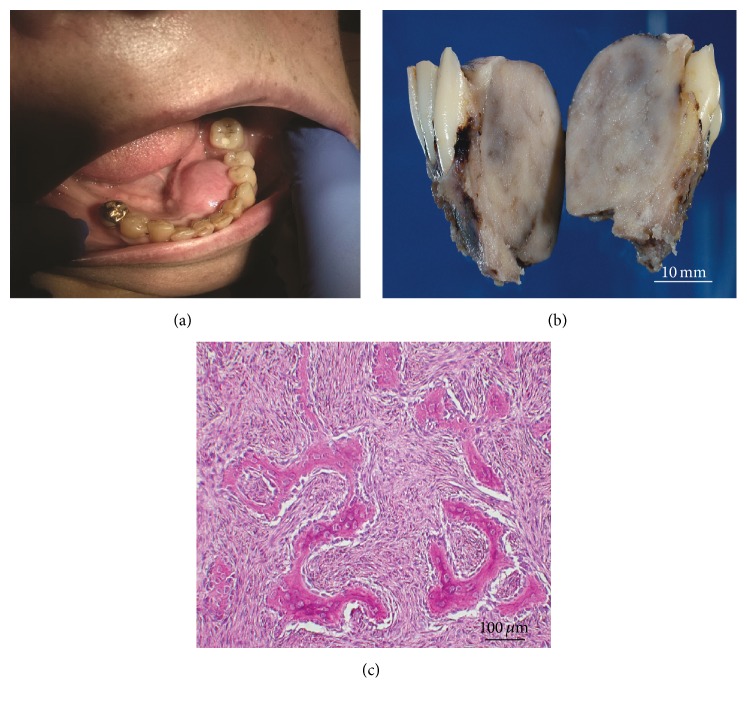
(a) View of the patient's oral cavity. (b) Cross-section through the partial mandibulectomy specimen, showing a well-delineated tumor pushing away the tooth roots. (c) Light microscopy shows a cellular fibrous stroma containing irregular woven bone trabeculae looking like Chinese letters, lined by many swollen osteoblasts, characteristic features of ossifying fibroma.

A 67-year-old Caucasian woman on chronic haemodialysis (HD) for 6 years for ESRD secondary to a crush syndrome developed a painful swelling in the floor of her mouth. Intraoral examination showed an oval-shaped solitary swelling with a maximal diameter of 2.5 cm. The lesion was white and hard and pushed the teeth forward (Figure 1(a)). The overlying skin and mucosa were intact. Regional lymph nodes were not palpable. Her past medical history included hypertension, hypercholesterolemia, and obesity. Her daily medications included Bumetanide 5 mg, Bisoprolol 5 mg, Pantoprazole 20 mg, Sevelamer 2400 mg, calcium polystyrene sulfonate 15 g 4 times/week, Epoetin Alfa 12000 units/week. Dialysis adequacy was optimal. Predialysis laboratory tests showed total calcium of 2.66 mmol/L (10.64 mg/dL) and phosphate of 1.70 mmol/L (5.26 mg/dL). Serum albumin and CRP levels were 43 g/L (Nl: 35–52) and 8 mg/L (<5), respectively. Intact parathyroid hormone, total alkaline phosphatase, and bone alkaline phosphatase concentrations were 4307 pg/mL (15–80), 1089 IU/L (35–105), and >120 ug/L (3–26 ug/L), respectively. There was no deficiency of 25 hydroxy vitamin D [36.2 ng/mL (30–100)]. Of note, ESRD was complicated by severe hyperparathyroidism for 4 years with PTH levels systematically above 2500 pg/mL and alkaline phosphatase levels ranging between 800 and 1500 IU/L. The patient repeatedly refused parathyroidectomy and cinacalcet intake. CA15.3 serum level was normal and mammography did not show any breast mass or localized microcalcifications. Chest X-ray was normal. There was no family history of hyperparathyroidism or neuroendocrine or jaw tumor. Computerized tomography of the jaw showed osteolysis of the anteroposterior side of the left mandibular, caused by a tumor surrounded, in some places, by a very thin cortical shell, creeping into and pushing forward dental roots 33, 34, and 35. Total body bone scintigraphy (Tc99m-diphosphonate hydroxyethylene) showed homogeneous increased uptake by the whole skeleton, especially by the sternomanubrial junction, with no other bone tumor. There was no increased uptake by the mandibular tumor. A parathyroid sestamibi scintigraphy revealed two nodules in the right and left inferior thyroid lobes, respectively.

The tumor was surgically removed (Figure 1(b)). Histological examination showed a cellular fibrous stroma containing irregular woven bone trabeculae, surrounded by an osteoblastic rim, a feature characteristic of an ossifying fibroma (OF) (Figure 1(c)). Currently, three years after resection, no recurrence of the OF is observed. The patient continues to refuse parathyroidectomy and cinacalcet intake.

## 3. Discussion

The patient presented an OF of the mandible. OF is part of the benign fibroosseous lesions (BFOL) that share similar histologic features, with replacement of normal bone by collagen and fibroblasts containing varying amounts of mineralized substance [1]. Radiologically, OF usually is a well demarcated hypodense image, surrounded by a sclerotic border, with internal small to large opacities. Histologically, OF is characterized by cellular fibrous stroma containing irregular woven bone trabeculae looking like Chinese letters, lined by a rim of swollen osteoblasts [2]. The most common sporadic forms mostly affect women between the second and fourth decades of life and develop in the jaw. Sporadic OF can present as a solitary or, rarely, as multiple lesions [3]. Its pathogenesis remains poorly understood. In contrast, hyperparathyroidism jaw tumor syndrome (HPT-JT) is associated with primary hyperparathyroidism and multiple OF of the mandible or maxilla. This inherited dominant autosomal condition, associated with a mutation of the tumor suppressor gene HRPT2 in chromosome 1q24–q32 encoding for a protein named parafibromin, is also characterized by the occurrence of parathyroid adenomas or carcinomas, Wilms tumor, renal cysts, renal hamartomas, and benign or malignant uterine tumors [3, 4].

The occurrence of an OF in a chronic HD patient is exceptional; to our knowledge, only three other cases have been published [5–7]. Although two patients were affected by severe uncontrolled hyperparathyroidism [PTH values between 1527 [6] and 2450 pg/mL [5]], the role of hyperparathyroidism on the pathogenesis of OF in the setting of end-stage renal disease remains unclear. Of note, there is no recurrence of OF three years after resection despite persistently high serum levels of intact PTH (above 3000 pg/mL). HPT-JT was very unlikely because the lesion was solitary and there was no family history of jaw tumor or endocrine disease.

After ruling out bone metastatic lesions, renal osteodystrophy in the context of severe secondary hyperparathyroidism was the most likely diagnosis, particularly as it may present as a solitary tumor in the oral cavity [8]. Brown tumors, a late manifestation of secondary hyperparathyroidism, are due to bone microfractures with subsequent bleeding causing localized osteoclast influx, osteolysis, and subsequent reactional medullar fibrosis and leading to the constitution of tumoral brown masses because of haemosiderin deposits [9]. In our patient, total body bone scintigraphy did not show increased uptake by the mandibular OF, an argument against a brown tumor. Of note, OF is composed of fibrocellular tissue containing mineralized material and osteoblasts and, unlike brown tumors, is characterized by the absence of osteoclasts and osteolysis. Typically, OF are painless. However our patient presented with a painful swelling in the floor of the mouth. We speculate that the pain was not caused directly by the OF but due to the fact that the OF pushed the tooth roots forward.

In conclusion, the occurrence of an OF in chronic HD patients is exceptional and most likely fortuitous. Surgical removal is the treatment of choice. Differential diagnosis must include bone tumoral lesions of the oral cavity, secondary to renal osteodystrophy. The potential role of severe, uncontrolled secondary hyperparathyroidism on its pathogenesis remains unclear. Parathyroidectomy may not be necessary to avoid its recurrence after surgical removal. Further studies are needed to elucidate the pathogenesis of OF and to determine the potential role of secondary hyperparathyroidism.
